# A novel use of biomechanical model-based deformable image registration (DIR) for assessing colorectal liver metastases ablation outcomes

**DOI:** 10.1002/mp.15147

**Published:** 2021-08-18

**Authors:** Brian M. Anderson, Yuan-Mao Lin, Ethan Y. Lin, Guillaume Cazoulat, Sanjay Gupta, A. Kyle Jones, Bruno C. Odisio, Kristy K. Brock

**Affiliations:** 1 Department of Imaging Physics, The University of Texas MD Anderson Cancer Center, Houston, Texas, USA; 2 The University of Texas Graduate School of Biomedical Sciences at Houston, Houston, Texas, USA; 3 Department of Interventional Radiology, The University of Texas MD Anderson Cancer Center, Houston, Texas, USA

**Keywords:** biomechanical model-based deformable image registration, colorectal cancer, colorectal liver metastases, liver ablation therapy

## Abstract

**Purpose:**

Colorectal cancer is the third most common form of cancer in the United States, and up to 60% of these patients develop liver metastasis. While hepatic resection is the curative treatment of choice, only 20% of patients are candidates at the time of diagnosis. While percutaneous thermal ablation (PTA) has demonstrated 24%–51% overall 5-year survival rates, assurance of sufficient ablation margin delivery (5 mm) can be challenging, with current methods of 2D distance measurement not ensuring 3D minimum margin. We hypothesized that biomechanical model-based deformable image registration (DIR) can reduce spatial uncertainties and differentiate local tumor progression (LTP) patients from LTP-free patients.

**Methods:**

We retrospectively acquired 30 patients (16 LTP and 14 LTP-free) at our institution who had undergone PTA and had a contrast-enhanced pre-treatment and post-ablation CT scan. Liver, disease, and ablation zone were manually segmented. Biomechanical model-based DIR between the pre-treatment and post-ablation CT mapped the gross tumor volume onto the ablation zone and measured 3D minimum delivered margin (MDM). An in-house cone-tracing algorithm determined if progression qualitatively collocated with insufficient 5 mm margin achieved.

**Results:**

Mann–Whitney U test showed a significant difference (*p* < 0.01) in MDM from the LTP and LTP-free groups. A total of 93% (13/14) of patients with LTP had a correlation between progression and missing 5 mm of margin volume.

**Conclusions:**

Biomechanical DIR is able to reduce spatial uncertainty and allow measurement of delivered 3D MDM. This minimum margin can help ensure sufficient ablation delivery, and our workflow can provide valuable information in a clinically useful timeframe.

## 1 | INTRODUCTION

Colorectal cancer (CRC) is a leading cause of cancer-related death worldwide^1^ and is the third most common cancer in the United States.^2^ A total of 25% of CRC patients present with colorectal liver metastasis (CLM) at the time of diagnosis, and up to 60% will develop CLM over their disease course.^3^ About two-thirds of deaths resulting from CRC are believed to be caused by CLM.^4^ While curative hepatic resection is the treatment of choice for patients presenting with CLM,^5^ only 20% of patients are candidates for surgery. For the remaining patients, systemic treatment or other forms of loco-regional therapy such as thermal liver ablation are offered.^6–9^ Liver ablation has demonstrated 5-year overall survival rates from 24% to 51%,^10–12^ and significantly longer overall survival when combined with systemic chemotherapy compared with chemotherapy alone.^13^

Minimal radiographic ablation margins have been described as one of the most relevant factors associated with improved local tumor control following thermal liver ablation,^14–18^ with minimum margins of 5–10 mm suggested to achieve optimal local control.^19,20^ Despite its criticality, ensuring a sufficient ablation margin can be challenging for several reasons. First, tumor visualization is limited on the intraprocedural noncontrast-enhanced computed tomography (CT) or ultrasound images for tumor targeting and probe guidance. Second, it is nearly impossible to distinguish between disease and ablated tissue in the post-treatment contrast-enhanced CT scan for hypoenhancing disease, preventing accurate measurement of delivered margin. Finally, accurate 3-dimensional (3D) assessment of ablation margin is difficult as rigid registration can be insufficient due to liver deformation throughout the procedure, and to the inherent inability to effectively assess ablation zone on a constrained intraprocedural time period without the aid of high-level computational analysis.

Current methods of ablation assessment often rely on 2D distance measurement to visible landmarks on the post-treatment imaging, do not ensure 3D minimum margin, or rely strictly on rigid registration.^21,22^ Rigid registrations can suffer in the presence of large deformation, and one recently published workflow has been shown to terminate when target registration errors greater than 3 mm are present,^22^ which commonly occur given liver deformations associated with patient’s breathing and changes in position,^23^ and post-ablation volumetric changes. While promising work leveraging a nonrigid intensity-based registration has been previously shown,^24,25^ “good” or “perfect” registration was achieved on only 62% of cases because of difference in liver position, difference in breathing phase, or artifacts in the image.

We hypothesized that the minimum ablation margin, calculated following the use of a biomechanical model-based deformable image registration (DIR), will differentiate patients with local tumor progression and those receiving sufficient ablation delivery. The biomechanical model-based registration proposed has demonstrated accuracy in the presence of liver position and breathing phase and is unaffected by artifacts as long as the liver boundary can be obtained.^26–29^ We also propose a method for establishing the relationship between the minimum ablation margin and the recurrence.

## 2 | MATERIALS AND METHODS

Data from 30 patients whose liver metastasis from colorectal cancer had been treated with local PTA at XXX were retrospectively evaluated under a prospectively maintained liver ablation registry approved by the institutional review board. Patients were included if they had a contrast-enhanced pre-treatment CT image within 45 days of PTA procedure, post-treatment contrast-enhanced portal-venous phase immediately performed at the end of PTA procedure, and follow-up imaging confirming freedom from local progression (mean 660 days) or evidence of local progression. The cohort comprised 14 patients with post-ablation local tumor progression (LTP) and 16 without LTP. Patient characteristics are listed in Table 1.

### 2.1 | Imaging data

Tri-phasic (arterial, portal-venous, delayed) CT images (minimum slice thickness, 2 mm; maximum slice thickness, 5 mm; minimum in-plane resolution, 0.5625 mm/pixel; maximum in-plane resolution, 0.8789 mm/pixel) were obtained with iodinated intravenous contrast injection prior and after to the ablation procedure for tumor identification and ablation zone assessment, respectively. Patients with biopsy-confirmed local progression also had their tri-phasic CT images at the time of recurrence diagnosis obtained.

**FIGURE 1:**
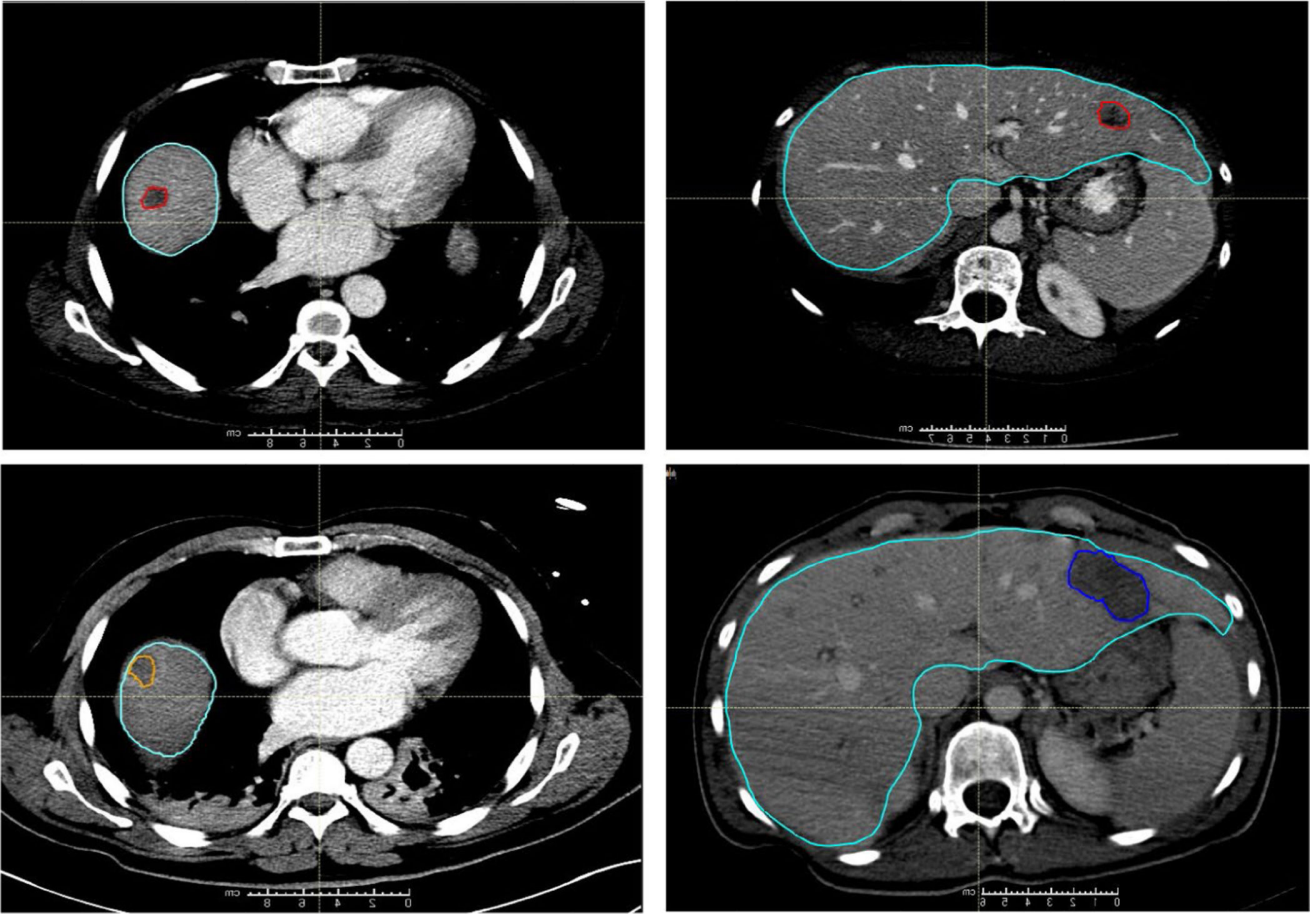
Computed tomography (CT) scans from two patients are shown, one in the left column and one in the right. (Top Row) Contouring of colorectal liver metastasis from diagnostic contrast-enhanced CT (red) and the liver contour (teal). (Bottom Row) Contouring of the ablation region (orange on the left and dark blue on the right) on the CT scan obtained immediately following the ablation

**FIGURE 2:**
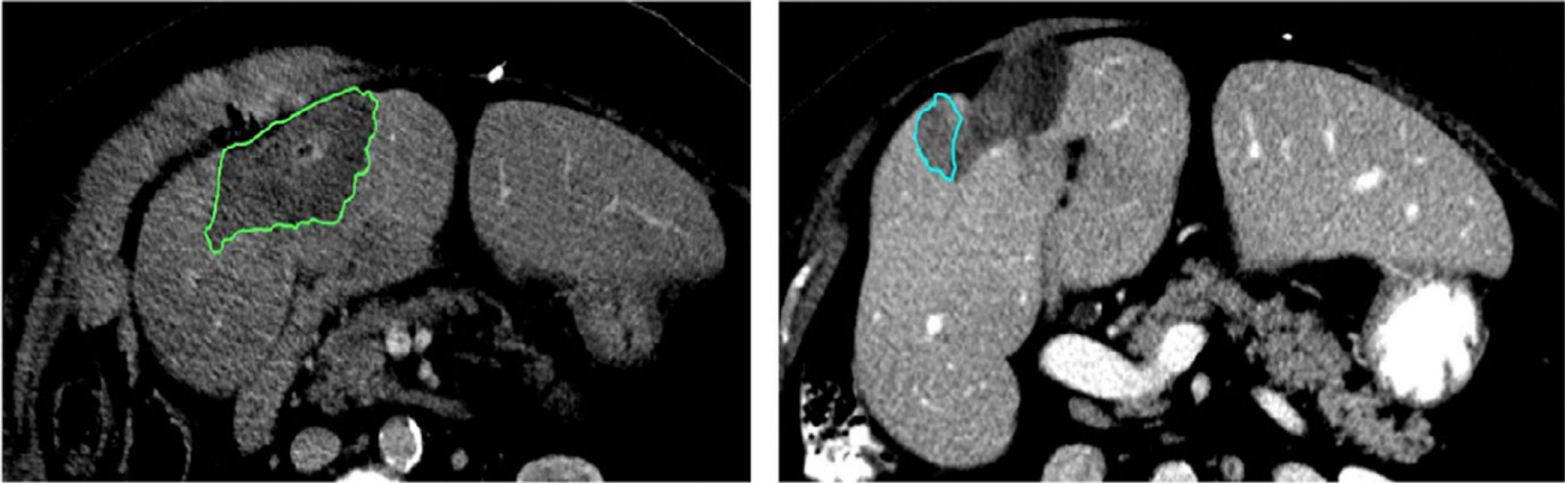
Left: Post-treatment ablation image with the ablation zone contoured in green. Right: Image from the same patient with recurrence contoured in blue. The difference in ablation zone volume from post-treatment to recurrence is 50%

All imaging data were uploaded into the FDA-approved radiation therapy treatment planning system (RayStation v5.0.2, RaySearch Laboratories). The liver was manually segmented on all portal-venous pre-treatment, intraprocedural guidance, post-treatment, and recurrence CT images. The venous phase of the pre-treatment tri-phasic CT was used to delineate the gross tumor volume (GTV) region of interest (ROI), as the contrast between normal tissue and disease was greatest in this phase. The post-treatment venous-phase CT image was used to define the ablation zone. The tumor recurrence was defined on the venous-phase CT. Segmentations of the GTV, ablation zone, and recurrent disease were performed under the guidance of a radiology-trained physician fellow and approved by a board-certified diagnostic and interventional radiologist with 11 years of experience in interventional oncology (Figure 1, 2).

### 2.2 | Biomechanical modeling

Two different registration techniques were performed to map the disease onto the post-treatment image to enable the calculation of the minimal ablation margin. First, rigid registration was performed between pre-treatment and post-treatment CTs using an automated gray-level cross correlation of the liver volume ROIs. Second, the biomechanical model-based DIR Morfeus, which is integrated with the RayStation system, was performed.^26,30^ Morfeus has been shown to have an accuracy on the order of the image voxel size when registering liver images for image-guided radiation therapy applications.^28,31^

Morfeus creates triangular surface meshes of the liver, which define the relationship between pre-treatment and post-treatment image sets. The optimization of two mesh creation parameters in RayStation was investigated in this study, the smoothing radius and triangular-mesh edge length. We investigated triangular-mesh edge values of 3, 6, 9, and 12 mm (default 6 mm) and smoothing radii of 1, 3, and 5 mm (default 1 mm). The metric of success for our selection of these parameters was the Dice similarity coefficient (DSC)^32^ between the generated triangular mesh and the manually defined slice-based contours on a patient-by-patient basis.

Morfeus then assigns Young’s modulus of 1000 Pa and Poisson’s ratio of 0.45, values which represent stiffness and compressibility, respectively, in a linear elastic material model. The correspondence of the generated triangular mesh determined the boundary conditions for the finite element analysis. The finite element analysis resulted in a dense deformation vector field, which was resampled to the ROI of the liver contour. The ablation zone was then propagated from the post-to pre-treatment image set using the results of the rigid registration only and the deformation vector field (Table 2).

### 2.3 | Assessing ablation margin

**FIGURE 3:**
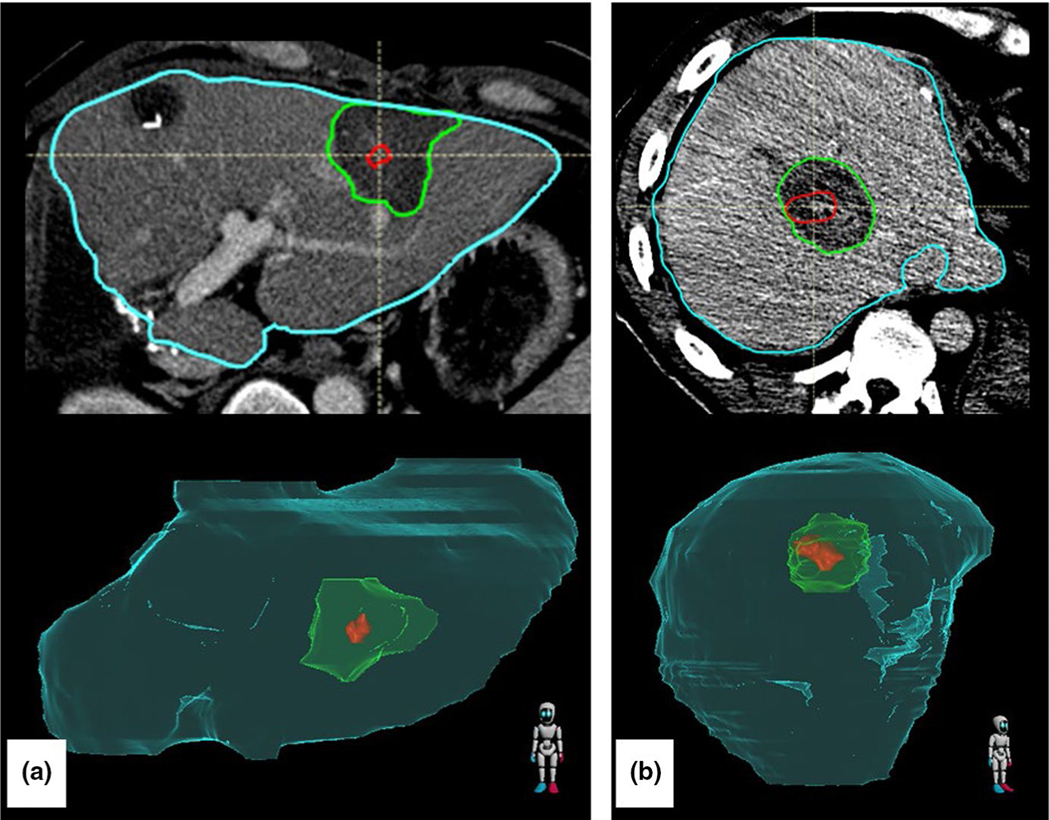
Two patients demonstrating the gross tumor volume (red) deformedly propagated onto the post-treatment scan with ablation zone (green) with the liver (teal). (a) Image shows a uniform surrounding of the disease by the ablation zone. (b) Image shows potentially insufficient ablation zone in the lateral aspect of the disease

With the disease contour mapped onto the registered post-treatment imaging, a 3D minimum distance to agreement (DTA) was computed between the GTV and ablation zone following rigid registration alone and rigid registration combined with biomechanical registration (Figure 3).

Regions in which the ablation zone contour entirely encompassed the GTV were considered to have positive ablation margins, and regions where the GTV and ablation zone contour overlapped had a margin of 0 mm. To remove the bias of minimum margin assessment when the GTV was on a subcapsular location, the ablation zone contour was expanded by 10 mm everywhere outside of the liver. This ensured that the minimum margin is accurately recorded as the minimum margin within the liver.

Comparison of rigid registration to deformable registration to distinguish locally recurring from nonlocally recurring patients was performed using a Mann–Whitney *U* test,^33^ as the data are not paired and not continuous since the minimum DTA has a close limit of 0 mm.

### 2.4 | Assessing recurrence

To quantify the correlation of 3D minimum margin with the location of tumor progression, registration between the post-ablation image and the recurrence image (obtained months later) is required, where physical changes in ablation cavity size challenge registration algorithms. Our data showed an average decrease of 37% in ablation cavity volume from post-treatment to recurrence images, likely due to dehydration of the tissue after thermal ablation and autophagy of the treated tissue.

A rigid registration, instead of a deformable registration, was performed to avoid the potential introduction of unknown errors, as there is no validated deformable registration available to account for ablation zone shrinkage.

A cone-tracing algorithm using spherical coordinates was used to assess correspondence of the recurrence to the 3D minimum ablation margin. Comparison of spherical coordinates requires that the ablation zone in both post-treatment and recurrence imaging be correctly registered translationally and rotationally. We developed a workflow to perform this focused rigid registration of the ablation CT and follow-up CT based on the identification and registration of vasculature in the normal liver within close proximity of the ablation region. While asymmetric shrinkage of the ablation zone in areas proximal to large vessels is assumed, our rigid registration is driven by similarities in the vessels to be robust to deviations in ablation zone recovery.

Liver vasculature was automatically segmented using an in-house vesselness algorithm^29,34^ on the post-treatment CT and the recurrence CT. A sphere of interest with a radius of 5cm, 7.5cm, and 10cm was defined on both images, centered on the ablation zone in the post-treatment CT and the recurrence CT. The rigid registration was then performed based solely on the vasculature within these spheres of interest. The 7.5-cm radius was chosen on the basis of visual inspection of the completed registration for all patients.

If sufficient vasculature could not be identified, manual registration was performed to best align the ablation zones on the post-treatment and recurrence images under the guidance of a trained interventional radiologist. To reduce bias, the radiologist was unaware of where the predicted minimum margin was located.

**FIGURE 4:**
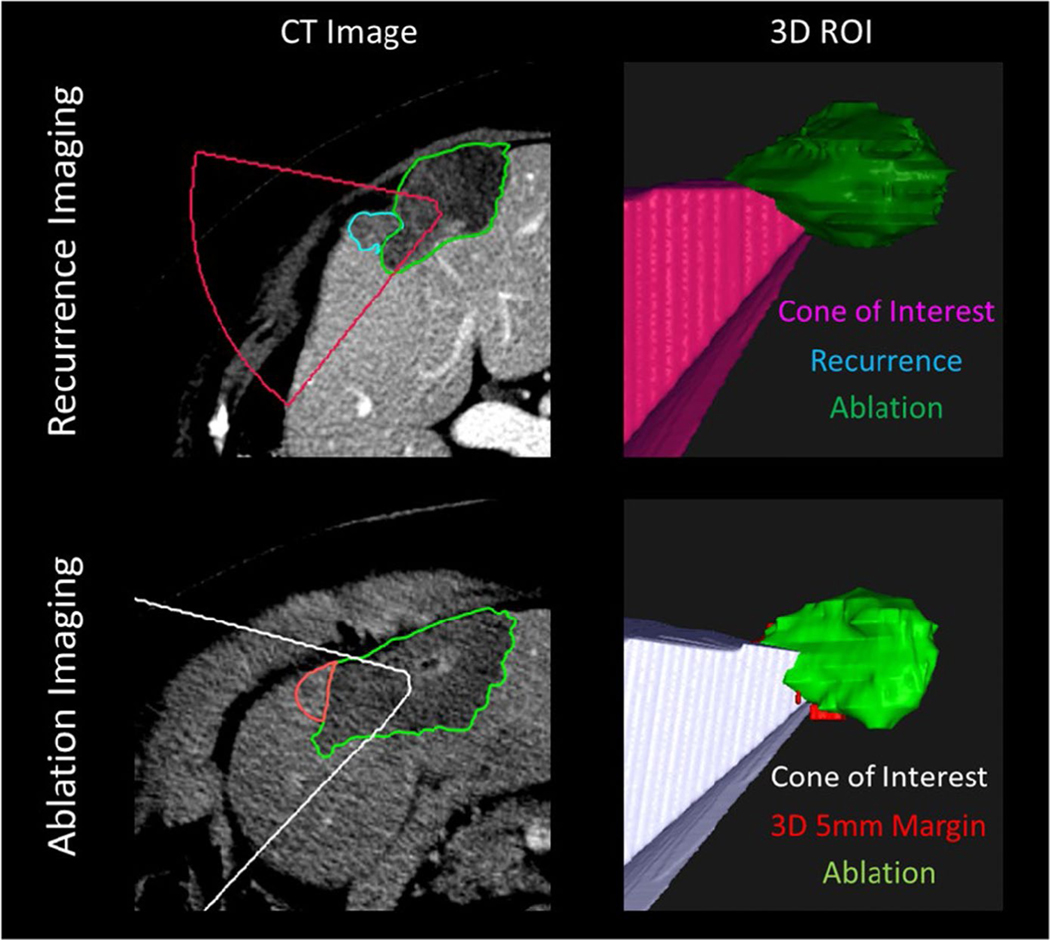
Top: Image of recurrence. Spherical coordinates of phi and theta intersecting the centroid of the recovered ablation zone (green) to each voxel of the recurrence (teal) are used to create a radiating cone (pink). Bottom: The recurrence phi and theta values are used to create another cone of interest (white) from the centroid of the post-treatment ablation zone (green) where the minimum margin has been identified (red). Because the cone intersects with the red 5-mm minimum margin ROI, we would claim that the minimum margin is in the same region that later turns into further progression

To create a quantitative metric of the relationship between the identified minimum margin on the post-treatment CT and the recurrence identified on the follow-up CT, we developed a 3D cone-tracing algorithm to determine if the spherical coordinates of the recurrence and the minimum ablation margin matched. Figure 4 illustrates the cone-tracing process, in which a 3D cone was created radiating from the center of the ablation zone on the recurrence CT, and the cone intersected every point of the contoured recurrence.

For visualization purposes, this cone was then mapped onto the centroid of the ablation zone on the post-treatment image. If the mapped cone overlapped with the minimum margin, the recurrence was considered to have occurred in the same relative region that the minimum margin existed. If the mapped cone did not overlap with the identified margin, the recurrence was considered to be unrelated to the minimum margin.

## 3 | RESULTS

### 3.1 | Assessing ablation margin

Rigid and deformable registrations between the pre-treatment images and both the intraprocedural and post-treatment images were successfully completed for all 30 patients.

**FIGURE 5:**
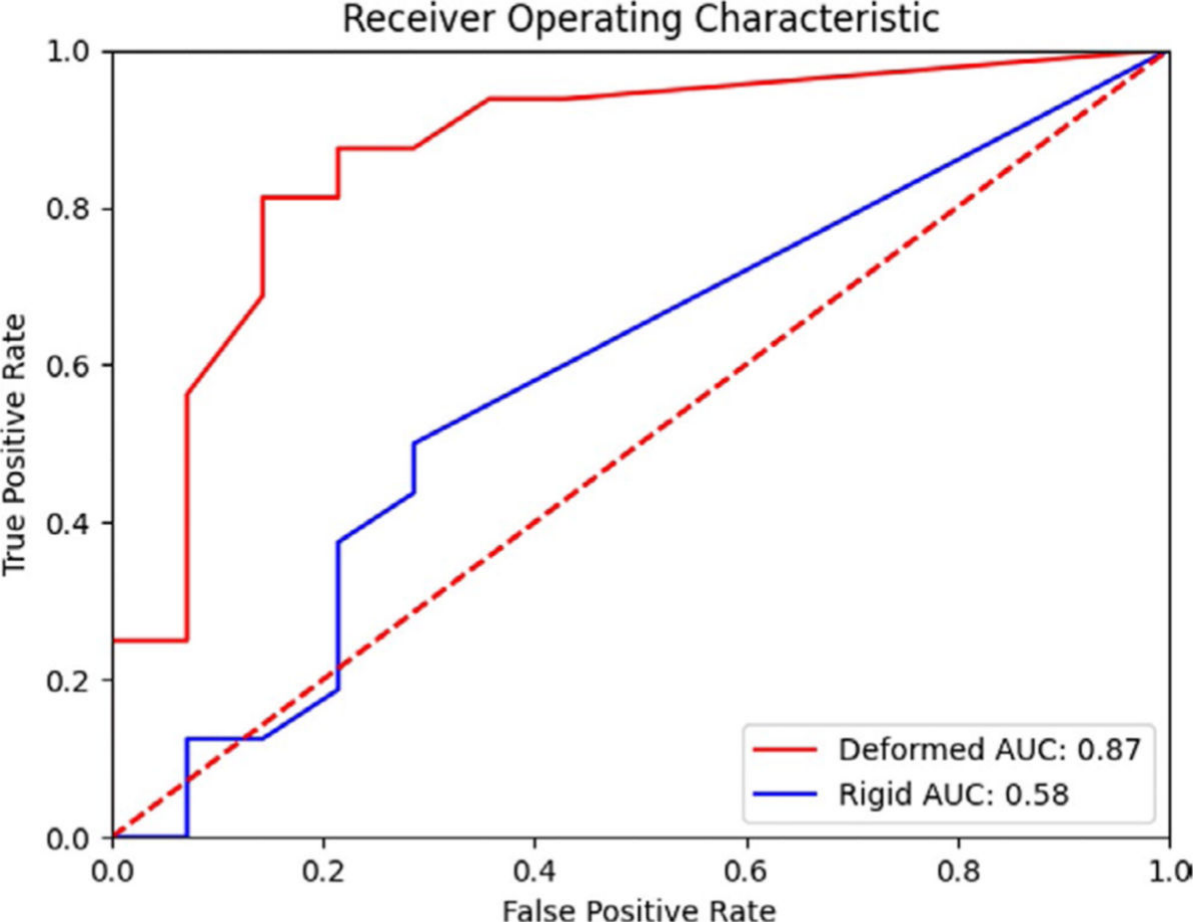
Receiver operator characteristic for identifying the development of local progression. The area under the curve (AUC) for the deformedly registered patients is 0.87, while for the rigidly registered patients is 0.58

For the deformable registration, on a patient-by-patient basis, the DSC between the generated triangular mesh and original contours was highest for a smoothing radius of 3 mm and a triangular-mesh edge length of 6 mm. The mean (min–max, median) minimum ablation margin around the GTV for patients without local recurrence was 3.19 mm (0.70–6.10, 2.90 mm), compared to patients diagnosed with local recurrence 1.14 mm (0–5.6, 0). Only three patients in the local recurrence group had margins greater than 2 mm. A Mann–Whitney *U* test revealed a significant (*p* < 0.01) difference between the two groups based on 3D minimum distance to agreement (Table S1). A receiver operating characteristic curve between both deformedly registered minimum margin and rigidly registered minimum margin can be seen in Figure 5.

Rigid registration resulted in considerable errors in the mapping of disease, where the mapped disease was shown to overlap within normal liver tissue. As the disease and ablation zone will appear hypoenhancing on portal-venous phase imaging, little to no amount of GTV volume mapped from the pre-treatment should be present outside of the ablation zone (barring small amounts due to difference in image sampling). We show the distribution of percentage GTV volume mapped outside of the ablation zone (should be 0) for both the rigid and deformable registrations (Table S1). The mean (std) of the percentage GTV volume mapped outside of the ablation zone for rigid registration was 19.51% (28.26%), while for deformable registration it was 1.39% (3.80%).

### 3.2 | Assessing recurrence

**FIGURE 6:**
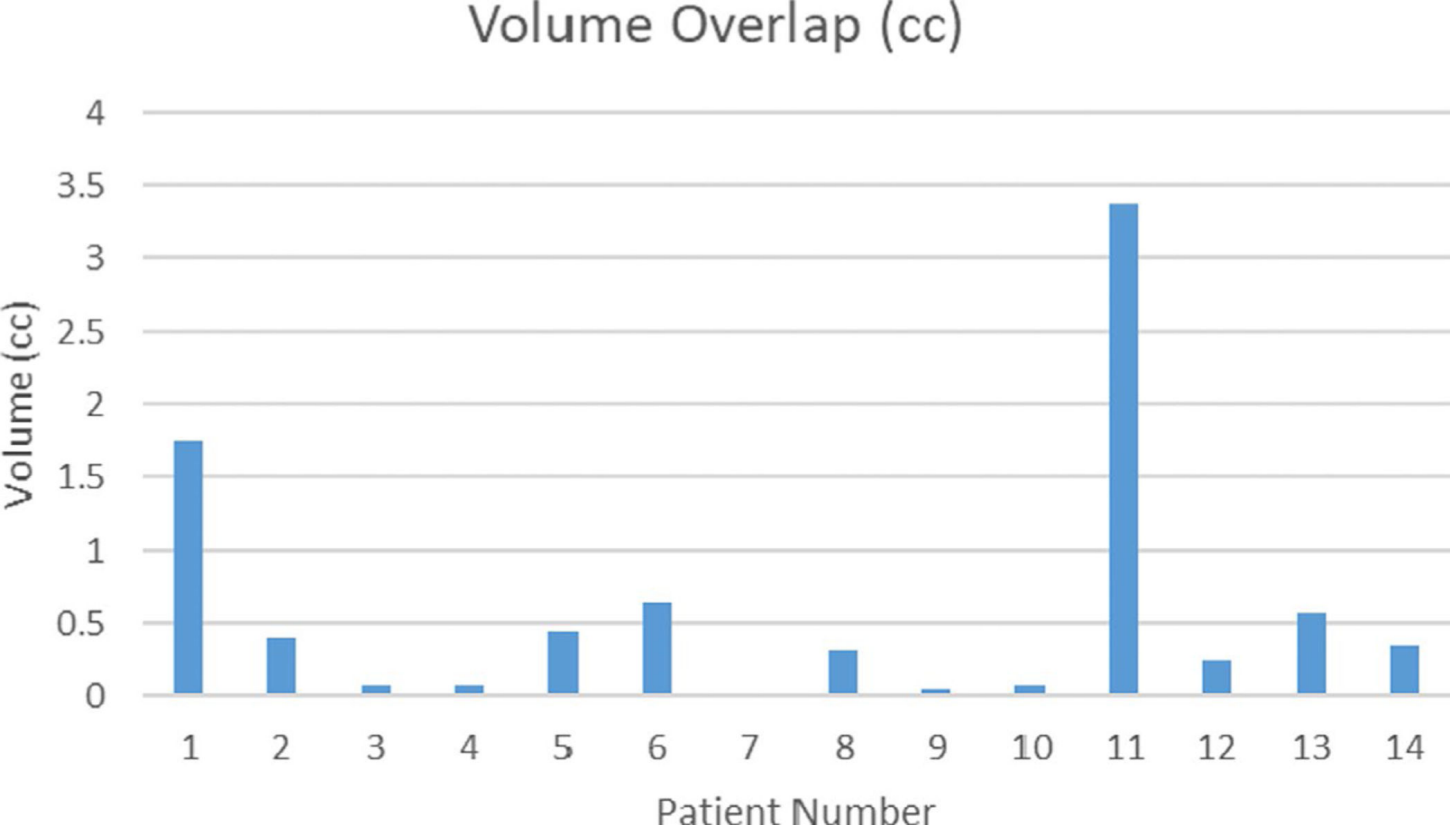
Amount of volume overlap (cc) between projected recurrence cone and 5 mm margin outside of ablation

In two cases, the contrast enhancement was insufficient to identify nearby vasculature, so manual rigid registration was performed focused on the ablation area. Of the 14 patients who experienced a recurrence, 13 (93%) had an overlap between the recurrence and the 5 mm expansion of disease outside of the ablation zone. That is, the recurrence occurred in the region where the ablation did not cover the intended 5 mm margin around the tumor. The median volume overlap between the projected cone and the minimum margin was 0.34 cc (range, 0–3.38 cc) (Figure 6).

**FIGURE 7:**
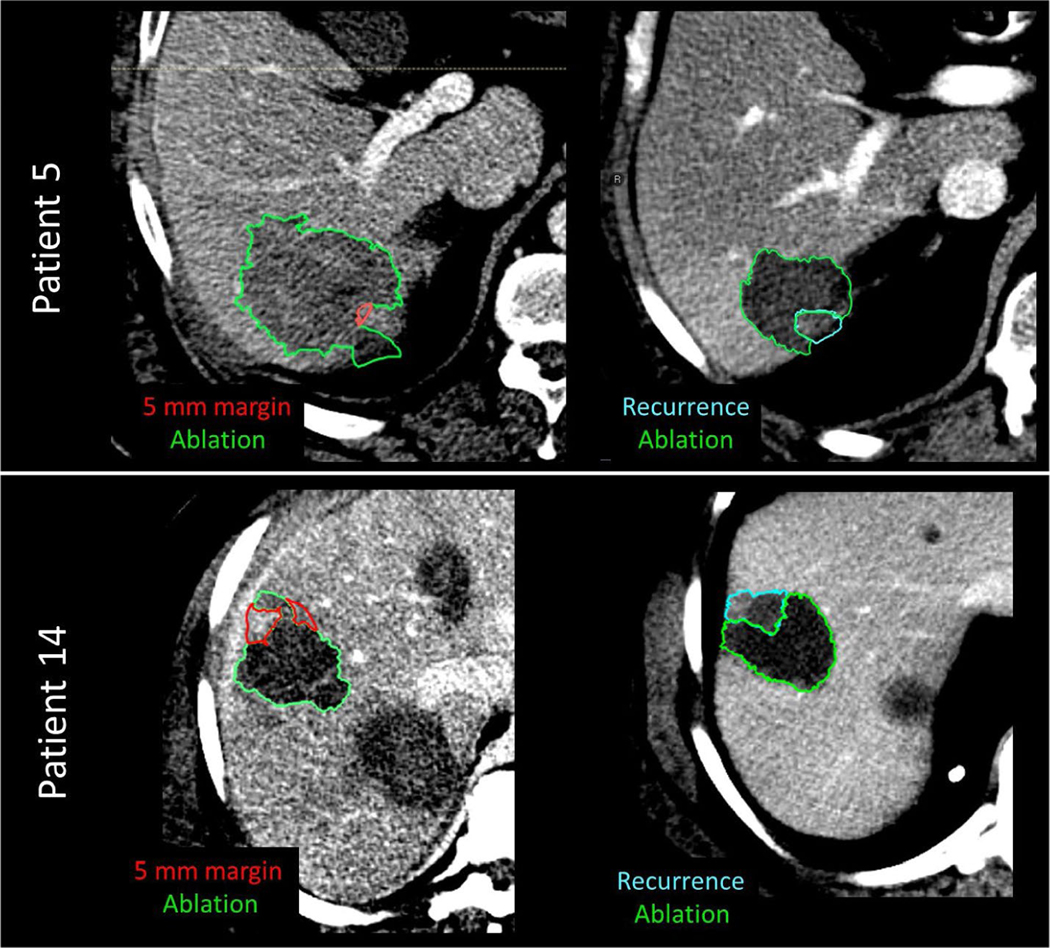
Example of 5-mm expansion on post-treatment CT images (left column) vs recurrence images (right column) for patient 5 and patient 14

Despite the fact that the volume of overlap can be very small, the consensus among our reviewing physicians is that the location of 5 mm expansion coincides with actual recurrence (Figure 7).

**FIGURE 8:**
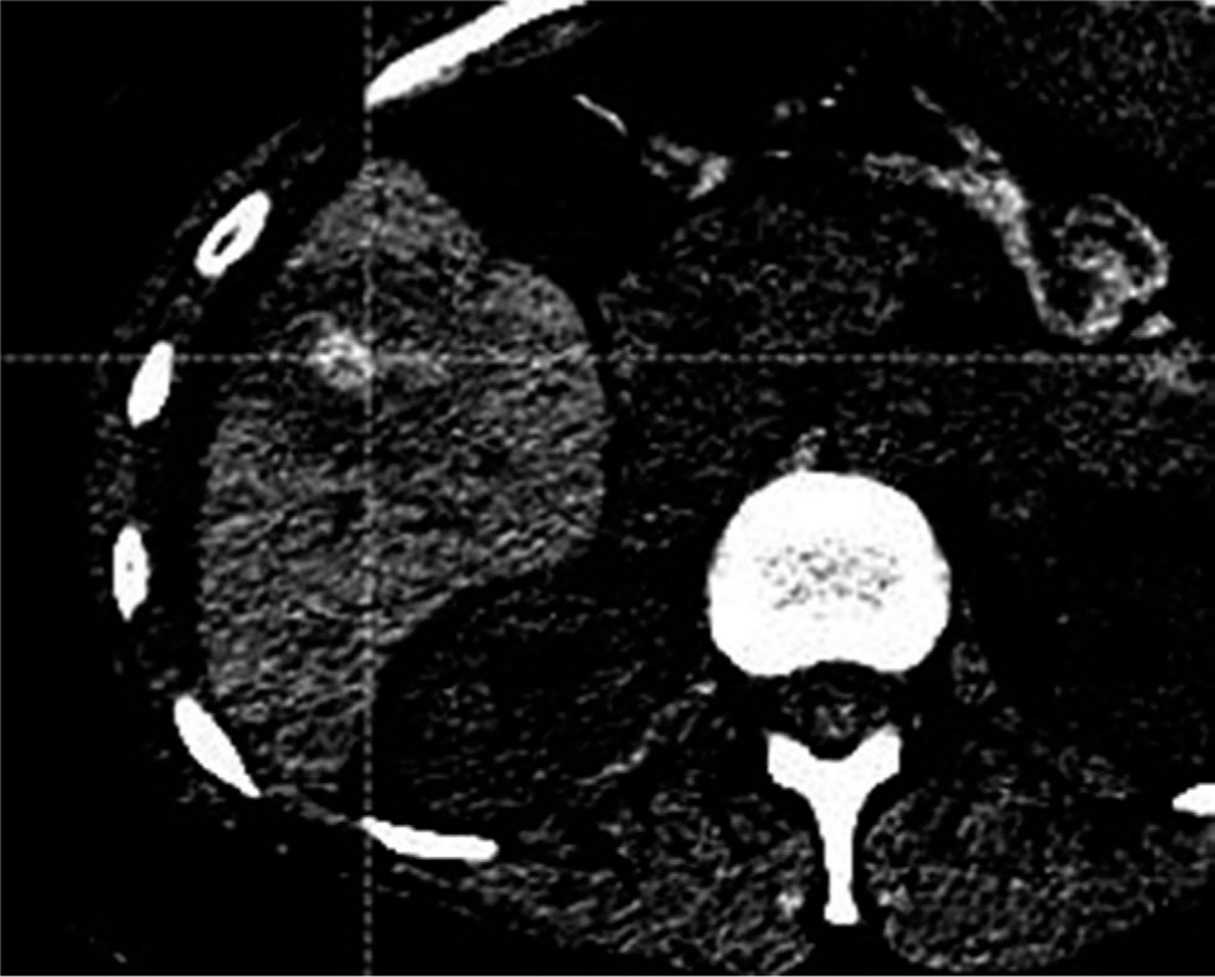
Patient 7, where no overlap between 5mm margin and actual progression was present. The poor contrast scan makes it difficult to identify where exactly the boundary of sufficient ablation is located

For patient #7, which did not have overlap, the definition of the ablation zone post-treatment was not very clear, with the scan appearing to be in the arterial phase, not the portal-venous (Figure 8).

## 4 | DISCUSSION

The efficacy of the proposed workflow was shown by distinguishing between the retrospectively obtained patients who experienced local recurrence from those who did not with high statistical significance using a Mann–Whitney *U* test (*p* < 0.01). We observed overlap between future recurrence and the minimum ablation margin in 86% of patients, and we believe this indicates that the minimum ablation margin is informative of where recurrence might develop. Our study demonstrated that the mean difference in minimum ablation margin between the group of patients who experienced local recurrence and the group who did not was large (2 mm, nearly 50% of the desired minimum margin for microwave therapy) and statistically significant (*p* < 0.01).

**FIGURE 9:**
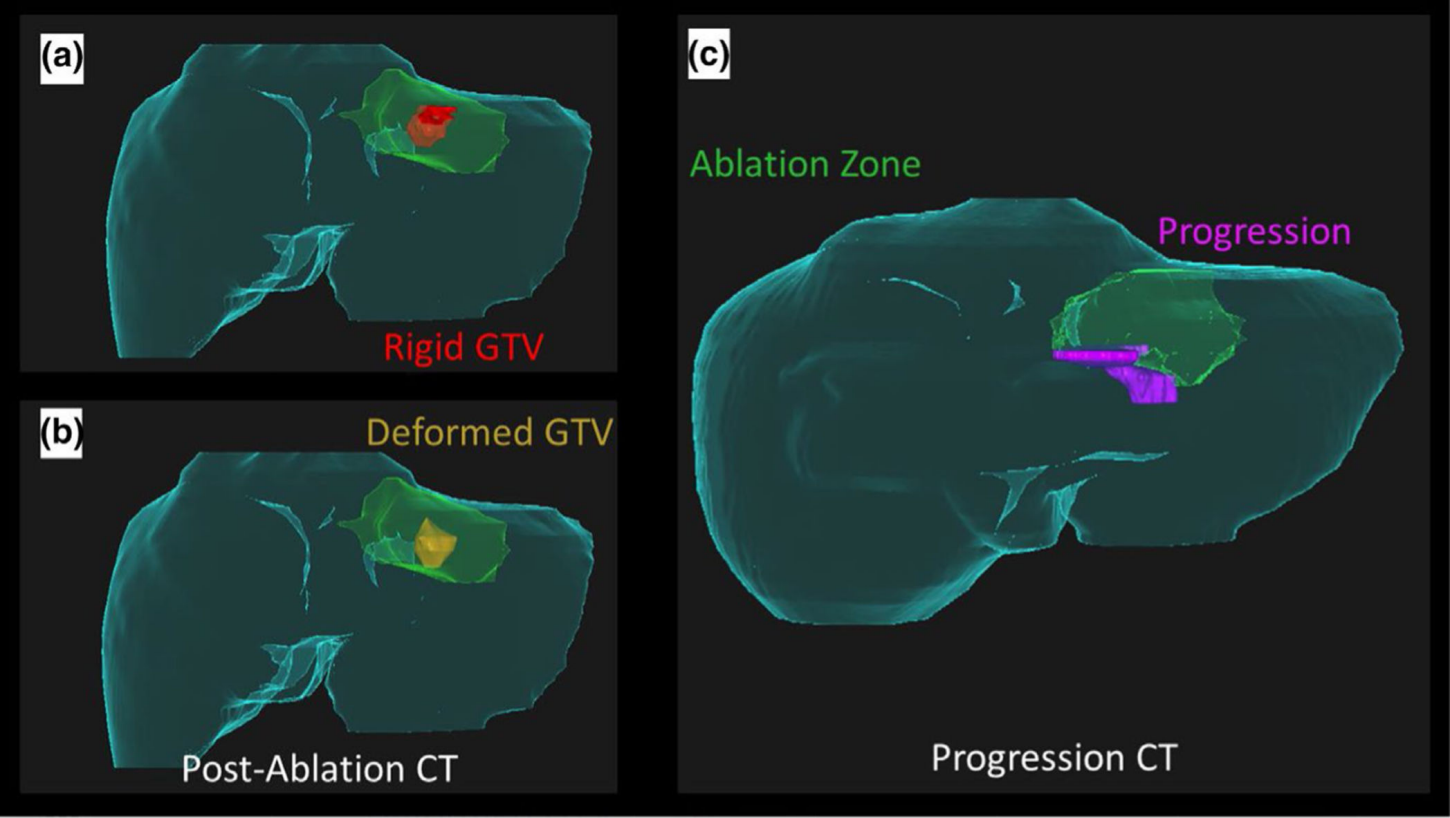
Failings of rigid registration. (a) Rigid registration of the gross tumor volume (GTV, red) onto the post-ablation CT maps part of the tumor outside of the ablation zone (green), and gives a false sense of confidence in the delivered margin caudally. (b) Deformable registration of the GTV (gold) onto the post-ablation CT indicates potentially insufficient ablation margin in caudal aspect. (c) Development of disease progression (pink) in the caudal aspect of the recovered ablation zone (green) corresponds with insufficient margin in (b)

Rigid registration alone was not sufficient to properly identify the minimum ablation margin. In many cases, the disease was mapped outside of the ablation zone entirely when rigid registration was used (Panel A, Figure 9). Most notably, patient 2 in the local recurrence group was recorded as having a minimum DTA of 5.3 mm via rigid registration, offering a false sense of confidence compared to the deformable registration (Panels B and C, Figure 9).

The proposed workflow has the potential to improve local control rates in patients, by assessing the minimum DTA immediately following ablation even in cases of deformation >1cm. The development of deep learning for liver segmentation has removed the previous challenges in clinical implementation introduced by manual segmentation.^35^ The process is fully automated after receiving segmentations of the GTV, ablation zone, and liver, with run times of approximately 5 min. The result returns the ablation margin on the pre-treatment image and an image showing the distribution of the ablation zone. This distribution can assist the physician in identifying where further ablation may be desired during the procedure. Previous work has shown that ablation assessment can reduce local progression rates, but required a secondary ablation procedure.^36^

In addition to determining the ablation margins, the algorithms proposed in this research can also be employed to ensure accurate placement of the probe into the tumor, especially in cases where contrast is no longer present in the image and artifacts from ablation probes impair tumor visibility. This would require a CT scan of the entire liver once the probe is inserted to perform biomechanical model-based DIR to map the tumor onto the image with the probe to evaluate the accuracy of placement.

Our work is primarily limited by the paucity of patients presenting with CLM who also had contrast-enhanced CT scans performed up to 45 days before treatment and post-treatment scans on the day of treatment. The amount of volume overlap between recurrence and volume of tissue within the 5 mm margin that was not covered by the ablation region also was small, less than 0.5 cc for 61% (8/13) of the patients in whom overlap was present. However, it is important to note that we expect that the overall volume of tissue that should have been included in the 5 mm ablation margin volume, but was not (i.e. area of missed ablation) would be very small, and therefore any overlap would also be small. However, it also demonstrates the potential importance of achieving a complete 5 mm ablation margin volume around the tumor.

Furthermore, our work is limited by the inherent variability in the segmentation of CLM and ablation zones. Under-estimation of the disease could lead to the belief of sufficient ablation delivery, while over-estimation of disease could lead to unnecessary ablation of normal tissues. This should also be considered for the use of this technology prospectively. Our results in the spatial overlap between the identified minimum margin and recurrence rely on the assumption that vessels near the recovering ablation zone are not distorted by relative differences in tissue regrowth during recovery.

## 5 | CONCLUSIONS

Our work demonstrates the potential utility of a biomechanical model-based DIR to aid in determining if sufficient ablation margin has been achieved and correlating the location of the recurrence to the minimum ablation area. For ease of use, we have created a GUI built into our treatment planning system which automates a majority of the process.

The clinical impact of these tools is currently being evaluated in a randomized phase II clinical trial.

## Supplementary Material

Supplementary Table

## Figures and Tables

**TABLE 1:** Patient characteristics

Characteristic	Local recurrence (N = 14)	Local control (N = 16)

Gender		
Male	8	11
Female	6	5
Age, y, mean (range)	60 (33–78)	57 (36–78)
Follow-up/recurrence^*^, d, mean (median, range)	209 (178, 64–554)	888 (660, 76–2501)

* Follow-up is the time from the procedure to recurrence or the last disease-free scan.

**TABLE 2:** Minimum distance to agreement (DTA) between the ablation contour and the tumor contour, % of GTV mapped volume and volume (cc) outside of ablation zone, for rigid and deformed registrations, stratified between patients with local recurrence and without local recurrence

Patient ID	Minimum DTA (mm)		% of GTV volume outside of ablation zone		GTV volume (cc) outside of ablation zone	

Local recurrence	Rigid	Deformed	Rigid	Deformed	Rigid	Deformed

1	0.00	0.00	91.30	4.00	1.05	0.25
2	0.53	0.07	2.74	0.00	0.05	0.00
3	0.00	0.10	0.66	0.00	0.02	0.00
4	0.25	0.00	0.00	0.00	0.00	0.00
5	0.00	0.05	24.95	0.00	1.03	0.00
6	0.24	0.00	16.90	0.58	0.52	0.01
7	0.00	0.00	2.39	3.47	0.03	0.04
8	0.00	0.47	18.07	0.00	0.56	0.00
9	0.00	0.00	70.96	17.65	1.58	0.37
10	0.00	0.17	11.68	0.00	0.21	0.00
11	0.00	0.00	17.10	0.21	0.57	0.01
12	0.00	0.00	68.01	12.01	3.99	0.48
13	0.10	0.00	0.00	0.00	0.00	0.00
14	0.00	0.24	11.09	2.50	0.18	0.05
Mean	0.08	0.08	23.99	2.89	0.70	0.09
Standard dev.	0.15	0.13	28.96	5.20	1.03	0.15

No local recurrence	Rigid	Deformed	Rigid	Deformed	Rigid	Deformed

1	0.40	0.60	49.16	0.00	0.34	0.00
2	0.00	0.61	100.00	0.00	0.96	0.00
3	0.27	0.24	0.00	0.00	0.00	0.00
4	0.00	0.48	2.72	0.00	0.06	0.00
5	0.17	0.20	0.00	0.00	0.00	0.00
6	0.10	0.45	0.00	0.00	0.00	0.00
7	0.15	0.28	0.00	0.00	0.00	0.00
8	0.00	0.30	35.24	0.39	3.20	0.04
9	0.24	0.45	0.00	0.00	0.00	0.00
10	0.00	0.52	41.19	0.89	1.10	0.03
11	0.07	0.24	0.00	0.00	0.00	0.00
12	0.00	0.07	0.00	0.00	0.00	0.00
13	0.00	0.14	14.10	0.00	0.06	0.00
14	0.20	0.20	0.00	0.00	0.00	0.00
15	0.00	0.30	2.32	0.00	0.04	0.00
16	0.00	0.00	4.86	0.00	0.06	0.00
Mean	0.11	0.34	15.60	0.08	0.36	0.00
Standard dev.	0.12	0.17	27.04	0.23	0.81	0.01

## Data Availability

Data available on request in compliance with IRB regulations.
