# Supplementary material for: A novel use of biomechanical model-based deformable image registration (DIR) for assessing colorectal liver metastases ablation outcomes
Source: Med Phys. Author manuscript; Available in PMC 2022 Aug 16. (PMC9380122; doi:10.1002/mp.15147)
Supplement: Supplementary Table [file NIHMS1821857-supplement-Supplementary_Table.docx]

Supplementary Table 1: Minimum Distance to Agreement (DTA) Between the Ablation Contour and the Tumor Contour, % of GTV mapped volume and volume (cc) outside of ablation zone, for Rigid and Deformed registration. Stratified Between Patients with Local Recurrence and without Local Recurrence.

| Patient ID | Minimum DTA (mm) | | % of GTV Volume outside of ablation zone | | GTV Volume (cc) outside of ablation zone | |
| --- | --- | --- | --- | --- | --- | --- |
| Local Recurrence | **Rigid** | **Deformed** | **Rigid** | **Deformed** | **Rigid** | **Deformed** |
| 1 | 0.00 | 0.00 | 91.30 | 4.00 | 1.05 | 0.25 |
| 2 | 0.53 | 0.07 | 2.74 | 0.00 | 0.05 | 0.00 |
| 3 | 0.00 | 0.10 | 0.66 | 0.00 | 0.02 | 0.00 |
| 4 | 0.25 | 0.00 | 0.00 | 0.00 | 0.00 | 0.00 |
| 5 | 0.00 | 0.05 | 24.95 | 0.00 | 1.03 | 0.00 |
| 6 | 0.24 | 0.00 | 16.90 | 0.58 | 0.52 | 0.01 |
| 7 | 0.00 | 0.00 | 2.39 | 3.47 | 0.03 | 0.04 |
| 8 | 0.00 | 0.47 | 18.07 | 0.00 | 0.56 | 0.00 |
| 9 | 0.00 | 0.00 | 70.96 | 17.65 | 1.58 | 0.37 |
| 10 | 0.00 | 0.17 | 11.68 | 0.00 | 0.21 | 0.00 |
| 11 | 0.00 | 0.00 | 17.10 | 0.21 | 0.57 | 0.01 |
| 12 | 0.00 | 0.00 | 68.01 | 12.01 | 3.99 | 0.48 |
| 13 | 0.10 | 0.00 | 0.00 | 0.00 | 0.00 | 0.00 |
| 14 | 0.00 | 0.24 | 11.09 | 2.50 | 0.18 | 0.05 |
| Mean | **0.08** | **0.08** | **23.99** | **2.89** | **0.70** | **0.09** |
| Standard Dev. | **0.15** | **0.13** | **28.96** | **5.20** | **1.03** | **0.15** |
| No Local Recurrence | **Rigid** | **Deformed** | **Rigid** | **Deformed** | **Rigid** | **Deformed** |
| 1 | 0.40 | 0.60 | 49.16 | 0.00 | 0.34 | 0.00 |
| 2 | 0.00 | 0.61 | 100.00 | 0.00 | 0.96 | 0.00 |
| 3 | 0.27 | 0.24 | 0.00 | 0.00 | 0.00 | 0.00 |
| 4 | 0.00 | 0.48 | 2.72 | 0.00 | 0.06 | 0.00 |
| 5 | 0.17 | 0.20 | 0.00 | 0.00 | 0.00 | 0.00 |
| 6 | 0.10 | 0.45 | 0.00 | 0.00 | 0.00 | 0.00 |
| 7 | 0.15 | 0.28 | 0.00 | 0.00 | 0.00 | 0.00 |
| 8 | 0.00 | 0.30 | 35.24 | 0.39 | 3.20 | 0.04 |
| 9 | 0.24 | 0.45 | 0.00 | 0.00 | 0.00 | 0.00 |
| 10 | 0.00 | 0.52 | 41.19 | 0.89 | 1.10 | 0.03 |
| 11 | 0.07 | 0.24 | 0.00 | 0.00 | 0.00 | 0.00 |
| 12 | 0.00 | 0.07 | 0.00 | 0.00 | 0.00 | 0.00 |
| 13 | 0.00 | 0.14 | 14.10 | 0.00 | 0.06 | 0.00 |
| 14 | 0.20 | 0.20 | 0.00 | 0.00 | 0.00 | 0.00 |
| 15 | 0.00 | 0.30 | 2.32 | 0.00 | 0.04 | 0.00 |
| 16 | 0.00 | 0.00 | 4.86 | 0.00 | 0.06 | 0.00 |
| Mean | **0.11** | **0.34** | **15.60** | **0.08** | **0.36** | **0.00** |
| Standard Dev. | **0.12** | **0.17** | **27.04** | **0.23** | **0.81** | **0.01** |
